# Non-response to rituximab therapy in rheumatoid arthritis is associated with incomplete disruption of the B cell receptor repertoire

**DOI:** 10.1136/annrheumdis-2018-214898

**Published:** 2019-06-19

**Authors:** Sabrina Pollastro, Paul L Klarenbeek, Marieke E Doorenspleet, Barbera D C van Schaik, Rebecca E E Esveldt, Rogier M Thurlings, Maria J H Boumans, Danielle M Gerlag, Paul P Tak, Koen Vos, Frank Baas, Antoine H C van Kampen, Niek de Vries

**Affiliations:** 1 Amsterdam Rheumatology and Immunology Center (ARC) | Department of Clinical Immunology and Rheumatology, Amsterdam UMC, location AMC, University of Amsterdam, Amsterdam, The Netherlands; 2 Department of Experimental Immunology | Amsterdam Infection & Immunity Institute, Amsterdam UMC, location AMC, University of Amsterdam, Amsterdam, The Netherlands; 3 Department of Clinical Epidemiology, Biostatistics and Bioinformatics, Amsterdam Public Health Research Institute, Amsterdam UMC, location AMC, University of Amsterdam, Amsterdam, The Netherlands; 4 Department of Rheumatology, Radboud University Medical Center, Nijmegen, The Netherlands; 5 Clinical Unit Cambridge, GlaxoSmithKline, Cambridge, UK; 6 Department of Medicine, Cambridge University, Cambridge, Massachusetts, USA; 7 Department of Rheumatology, Ghent University, Ghent, Belgium; 8 Flagship Pioneering, Cambridge, Massachusetts, USA; 9 Laboratory for Diagnostic Genome Analysis, Leiden University Medical Center, Leiden, The Netherlands

**Keywords:** rheumatoid arthritis, rituximab, b cells, b-cell receptor repertoire, next-generation sequencing

## Abstract

**Objective:**

To gain more insight into the dynamics of lymphocyte depletion and develop new predictors of clinical response to rituximab in rheumatoid arthritis (RA).

**Methods:**

RNA-based next-generation sequencing was used to analyse the B cell receptor (BCR) repertoire in peripheral blood and synovial tissue samples collected from 24 seropositive patients with RA treated with rituximab. Clonal expansion, mutation load and clonal overlap were assessed in samples collected before, at week 4 and at week 16 or 24 after treatment and correlated to the patients’ clinical response.

**Results:**

After 4 weeks of rituximab-induced B cell depletion, the peripheral blood BCR repertoire of treated patients consisted of fewer, more dominant and more mutated BCR clones. No significant changes in the synovial tissue BCR repertoire were detected until week 16 post-treatment, when a reduced clonal overlap with baseline and an increased mutation load were observed. In patients who were non-responders at month 3 (n=5) using the European League Against Rheumatism response criteria, peripheral blood samples taken at week 4 after rituximab treatment showed more dominant clones compared with moderate responders (n=9) (median (IQR): 36 (27–52) vs 18 (16–26); p<0.01) and more clonal overlap with the baseline (median (IQR): 5% (2%–20%) vs 0% (0%–0%); p≤0.01).

**Conclusion:**

Significant changes in BCR clonality are observed in peripheral blood of patients 4 weeks after rituximab treatment, while changes in synovial tissue were observed at later time points. Incomplete depletion of the dominant baseline peripheral blood BCR repertoire in the first month of treatment might predict clinical non-response at 3 months.

**Key messages**

**What is already known about this subject?**

- Rituximab induces deep depletion of the B cells, but up to 35% of treated patients with rheumatoid arthritis (RA) do not achieve clinical European League Against Rheumatism response (Randomized Evaluation of Long-Term Efficacy of Rituximab in RA (REFLEX) trial).
- Prediction of response to this treatment remains challenging.

**What does this study add?**

- By analysing for the first time the B cell receptor (BCR) repertoire in peripheral blood and synovial tissue samples of patients with RA before and at different time points after treatment with rituximab, we provide new insight into the spatial and temporal effects of this drug on the B cell compartment.
- We observed major changes in BCR clonality at 4 weeks after treatment, while the same effects are observed in synovial tissue only at later time points.
- We found a correlation between incomplete depletion of the pretreatment BCR repertoire in peripheral blood after 1 month of treatment and worse treatment response evaluated at 3 months after treatment.

**How might this impact on clinical practice or future developments?**

- The early identification of non-responder patients based on early BCR clonality changes would allow clinicians to timely switch patients who are unlikely to respond to rituximab treatment to other therapies.

## Introduction

Rheumatoid arthritis (RA) is a chronic autoimmune disease that affects 1% of the population worldwide. The disease aetiology is not completely understood, and current treatments have limited efficacy.^1 2^ Currently, patients are treated long term with immunosuppressive therapies to control inflammation associated with pain, disability and joint destruction if left untreated. Understanding the mechanism underlying effective drug treatment may help to improve the response to therapy.

During the last decades, the introduction of biologicals constituted a major step forward in the treatment of RA. From a research perspective, the use of targeted therapies offers the opportunity to study the changes in the immune system while one cellular or molecular component is temporary depleted from the system. Rituximab, a chimeric monoclonal antibody directed against the B cell surface molecule CD20, is an example of such a targeted therapy. A single treatment with rituximab induces >98% depletion of the B cells in peripheral blood lasting for at least 4–5 months, but clinical response is achieved in only 50%–65% of treated patients.^3 4^

The exact mechanism by which depletion of CD20-bearing B cells leads to clinical improvement is still unclear.^5^ Incomplete B cell depletion in peripheral blood after rituximab treatment was initially considered the likely explanation for treatment failure.^6–8^ This notion is supported by the observation that high-sensitivity flow cytometry analyses could still detect class-switched memory B cells and plasma cell precursors in peripheral blood in more than half of the patients after the first rituximab infusion.^9^ However, a clear correlation between the degree of B cell depletion in the peripheral blood and clinical response was not convincingly demonstrated.^10^ A second hypothesis to explain treatment failure is that this drug does not deplete all relevant B cell subsets, in particular in the tissues where B lineage cells may be protected against undergoing cell death.^4^ It has been consistently shown that B lineage cells may be protected from rituximab-induced depletion in the tissues, including synovial tissue and bone marrow.^11 12^ Of interest, there is a significant relationship between changes in B cell-derived plasma cells in the synovium and subsequent clinical response.^12^

Studies have been limited so far to the use of phenotypic cell markers. The development of RNA-based next-generation sequencing allowed us for the first time to analyse the B cell receptor (BCR) repertoire in different body compartments before and at different time points after rituximab treatment. The results presented here provide new insights into the effects of rituximab in peripheral blood and synovial tissue over time, and may help to identify new biomarkers predictive of response to treatment that could be used to optimise treatment response in the future.

## Methods

### Patients

Twenty-four patients with active, IgM-rheumatoid factor and/or anti-cyclic citrullinated peptides 2 test (CCP2)-positive RA^13^ were included in this study. Study inclusion criteria, patient characteristics and clinical trial design have been described extensively before.^12 14^ The baseline characteristics of patients are reported in online supplementary table S1. The treatment protocol consisted of two intravenous injections of 1000 mg rituximab (Roche, Woerden, The Netherlands) on days 1 and 15. Premedication with methylprednisolone was not allowed since it could have confounded the study, but stable methotrexate, prednisone and non-steroidal anti-inflammatory drugs were allowed. Patients were assessed for disease activity using the Disease Activity Score 28 joints.

10.1136/annrheumdis-2018-214898.supp1 Supplementary data

### Samples

Peripheral blood and synovial tissue samples were collected before treatment (baseline (FU0)), at week 4 (follow-up 1 (FU1)) and at week 16 (follow-up 2 (FU2)) after rituximab treatment, as described before.^12 14^ For some patients the week 16 post-treatment peripheral blood sample was not available and peripheral blood samples collected at week 24 were analysed instead. Peripheral blood mononuclear cells (PBMCs) were isolated from total blood using Ficoll separation (GE Healthcare, ref 17-1440-02) and cryopreserved in liquid nitrogen until RNA isolation. Synovial tissue biopsies were collected by needle arthroscopy as previously described^15^ from an inflamed knee as judged by the treating physician and cryopreserved in liquid nitrogen until RNA isolation.

### Linear amplification and next-generation sequencing of BCR repertoires

Starting material for RNA extraction consisted of 10 million PBMCs for peripheral blood samples and homogenised synovial tissue biopsies for synovial tissue samples. RNA extraction, complementary DNA synthesis and quantitative amplification of BCR heavy chain molecules were performed as previously described.^16–18^ Information on the number of total and unique obtained sequences is reported in online supplementary table S2. Data sets were equalised to the minimum number of reads retrieved per tissue origin (2462 reads for peripheral blood samples, 2558 for synovial tissue samples). Sequences with 100% CDR3 identity at the amino acid level were regarded as a ‘BCR clone’, and the frequency of each BCR clone was calculated as the number of identical sequences divided by the total sequences retrieved in each sample. BCR clones with a frequency above 0.5% were termed ‘dominant clones’. Additional analysis on the reproducibility of our approach and impact of sequencing depth on the quantification of dominant clones is reported in online supplementary figures S2 and S3.

### Statistics

Data are presented as mean and SD or median and IQR after D’Agostino and Pearson omnibus test for normality. Differences between groups were evaluated using Mann-Whitney U test for unpaired data, one-way analysis of variance or Kruskal-Wallis test of ranks data; p values less than 0.05 were considered statistically significant. Prism V.7 software (GraphPad, San Diego, California) and RStudio (R V.3.3.2) were used to perform the analysis.

## Results

### Rituximab treatment induces clonality changes of the BCR repertoire in peripheral blood but not in synovial tissue

Following rituximab infusion there was deep depletion of CD19+ B cells in peripheral blood.^12^ As a first step we therefore tested whether our RNA-based technology was sensitive enough to detect the BCR repertoire in B cell-depleted peripheral blood samples. In all patients analysed, we were able to detect the BCR repertoire of both post-treatment peripheral blood samples (week 4 and week 16/24; online supplementary figure S1).

**Figure 1:**
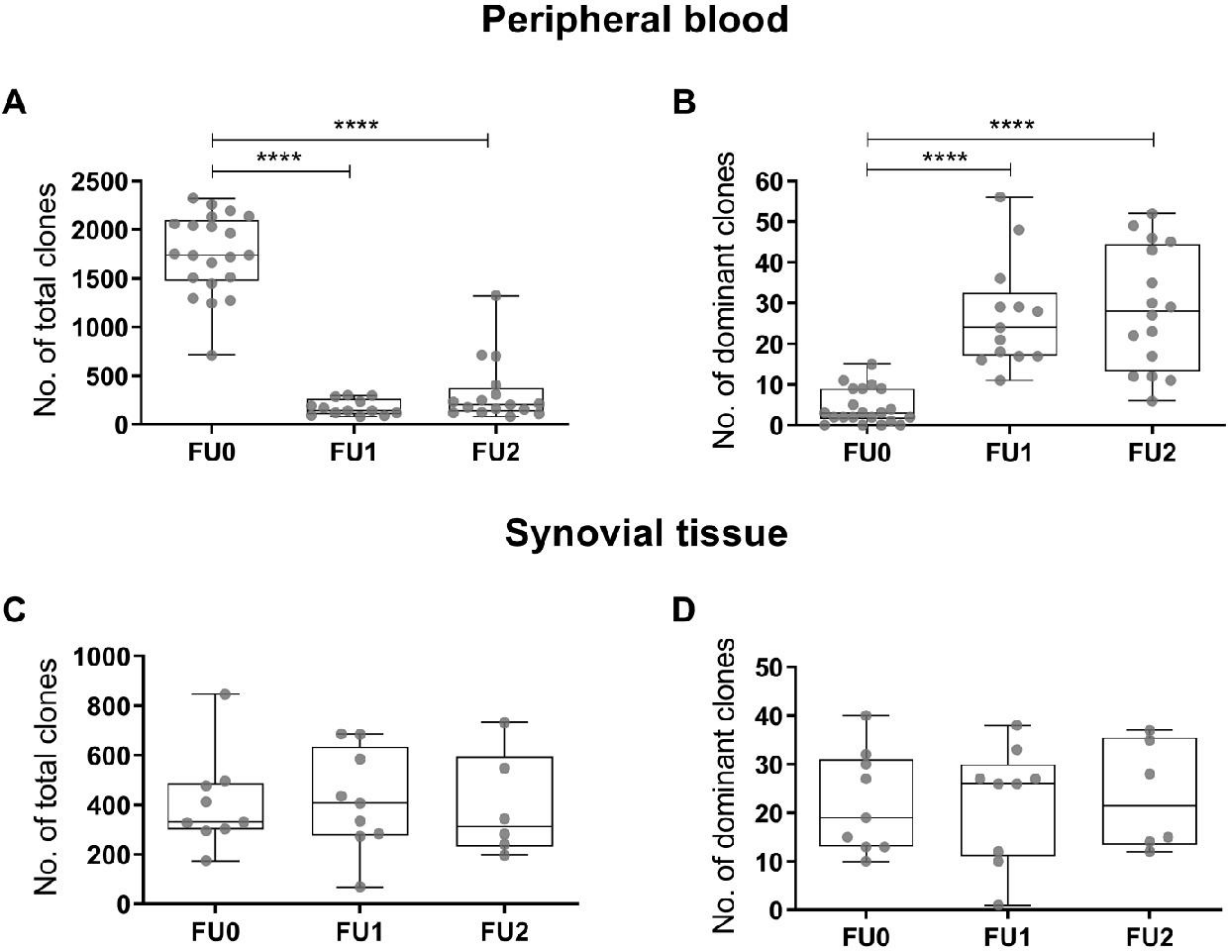
Peripheral blood and synovial tissue BCR repertoire clonality during rituximab treatment. (A–B) Boxplots showing the number of total BCR clones and the number of dominant BCR clones (clonal frequency >0.5%) in the peripheral blood repertoire before (FU0=baseline) and after (FU1=week 4; FU2=week 16/24) treatment with rituximab. (C–D) Boxplots showing the number of BCR clones and the number of dominant BCR clones (clonal frequency >0.5%) in the synovial tissue repertoire before (FU0=baseline) and after (FU1=week 4; FU2=week 16/24) treatment with rituximab. Boxplots show the median and 25th and 75th interquartile, error bars show the range, and single data points are depicted in grey (****p≤0.0001 using two-way analysis of variance). BCR, B cell receptor.

Next, we evaluated the effects of rituximab treatment on the BCR repertoire in both peripheral blood and synovial tissue. To this end we compared the number of BCR clones and the number of dominant BCR clones (clonal frequency >0.5% of the total repertoire) in samples collected before and after therapy. The number of BCR clones in the peripheral blood repertoire was significantly decreased after rituximab treatment compared with pretreatment conditions (median (IQR): FU0: 1738 (1476–2095); FU1: 141 (105–206); FU2: 206 (131.0–379); p<0.0001), while the number of dominant BCR clones was significantly increased (median (IQR): FU0: 3 (2–9); FU1: 24 (17–33); FU2: 28 (13–45); p<0.0001) (figure 1A,B). In contrast, there were no significant changes in the number of BCR clones or in the number of dominant BCR clones in the synovium at the different time points after treatment (figure 1C,D). Taken together these data indicate that rituximab drastically changes the peripheral blood BCR repertoire clonality while this was not observed in the BCR repertoire in the synovial tissue samples from the same patients at the same time points.

### The postdepletion BCR repertoire is mainly composed of highly mutated BCR clones

**Figure 2:**
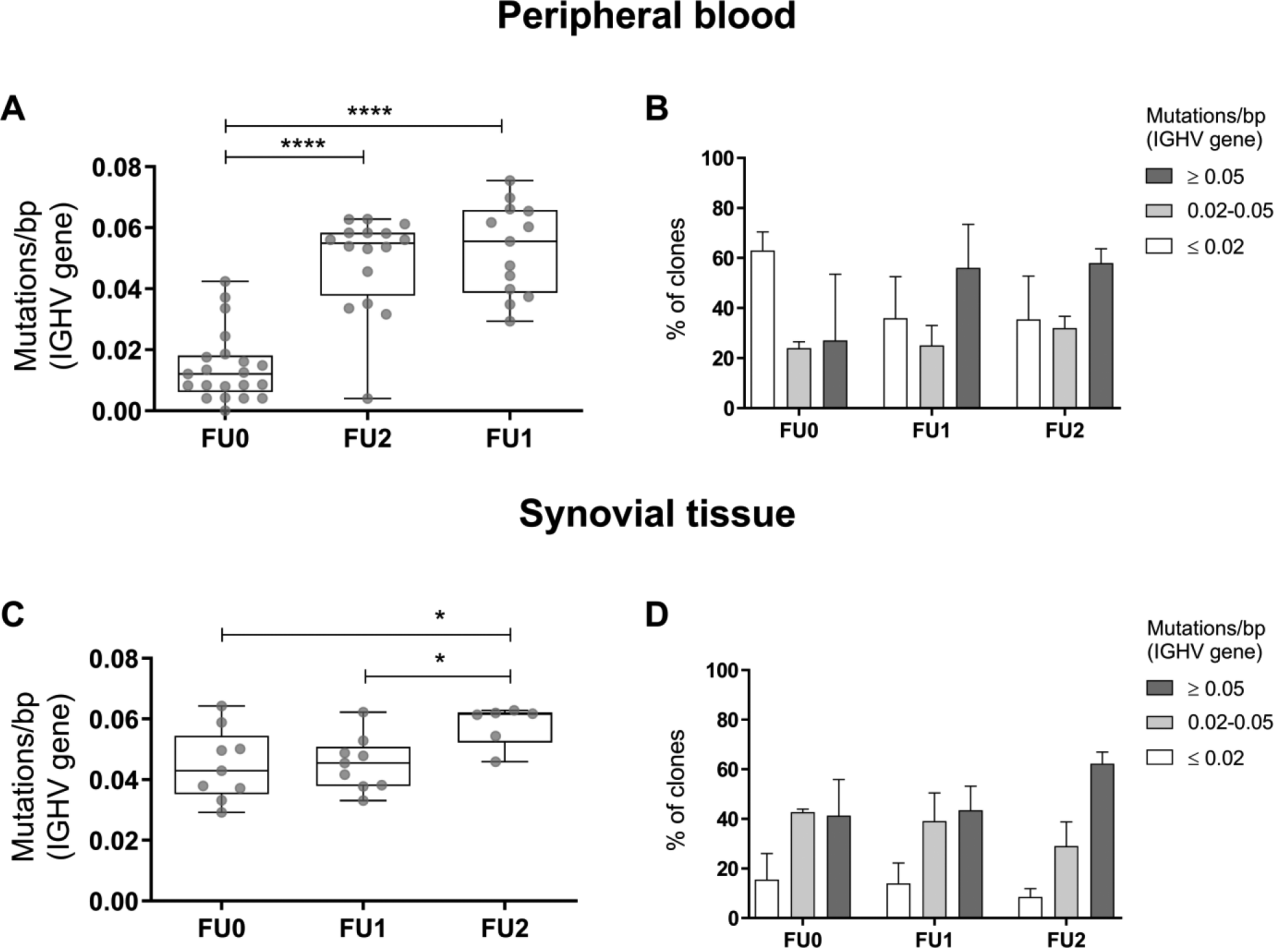
Mutation analysis in peripheral blood and synovial tissue BCR repertoire during rituximab treatment. (A and C) Boxplots showing the mutation load (expressed as mutations/bp) in the peripheral blood (A) and synovial tissue (C) BCR repertoire before (FU0=baseline) and after (FU1=week 4; FU2=week 16/24) treatment with rituximab. Boxplots show the median and 25th and 75th interquartile, error bars show the range, and single data points are depicted in grey (*p≤0.05, ****p≤0.0001 using two-way analysis of variance). (B and D) Bar plots showing the distribution of BCR clones with low (≤0.02 mutations/bp, white bars), medium (0.02–0.05 mutations/bp, light grey bars) and high (≥0.05 mutations/bp, dark grey bars) mutation load in the peripheral blood (B) and synovial tissue (D) repertoire before (FU0=baseline) and after (FU1=week 4; FU2=week 16/24) treatment with rituximab. Bars’ height shows the median and error bars show the IQR. BCR, B cell receptor.

The phenotypic characterisation of the postdepletion B cell compartment using conventional flow cytometry is hindered by the small number of cells that can be detected, but high-sensitivity flow cytometry did allow detection of rituximab-residual B cells in peripheral blood.^9^ To gain more insight into the phenotypic composition of the postdepletion BCR repertoire, we analysed the mutation load in the BCR variable heavy (immunoglobulin heavy chain variable (IGHV)) genes as an indication of the maturation status of the total BCR repertoire. A high mutation load indicates that the repertoire is dominated by mature BCR clones (ie, memory and plasma blasts/cells), while a low mutation load indicates that the BCR repertoire is mainly composed of immature BCR clones (ie, naïve). When we compared the mutation load (expressed as mutations/base pairs (bp)) in the peripheral blood repertoire of samples collected before and after rituximab treatment, we found that the posttreatment repertoire had a significantly higher mutation load at both time points after treatment compared with the pretreatment repertoire (median (IQR): FU0: 0.012 (0.006–0.018); FU1: 0.056 (0.039–0.066); FU2: 0.055 (0.038–0.058); p<0.0001). In synovial tissue, there was a higher mutation load compared with peripheral blood before treatment. We could demonstrate a further increase in the mutation load in the synovial tissue samples taken 16 weeks after treatment (median (IQR): FU0: 0.043 (0.035–0.054); FU1: 0.045 (0.038–0.051); FU2: 0.062 (0.052–0.062); p=0.0325) (figure 2A, C). We further characterised the phenotypic composition of the BCR repertoire before and after treatment by evaluating the fraction of repertoire composed of BCR clones with low (≤0.02 mutations/bp), medium (0.02–0.05 mutations/bp) and high (≥0.05 mutations/bp) mutation load (figure 2B, D). The peripheral blood BCR repertoire before treatment is mainly composed of BCR clones with low mutation load (mean percentage 59%), while the highly mutated BCR clones dominate the post-treatment repertoire at both time points analysed (mean percentage FU1: 57%; mean percentage FU2: 58%). In synovial tissue, however, medium and highly mutated BCR clones already dominate the repertoire before treatment (mean percentage medium: 39%; high: 45%), and a further increase in fraction of highly mutated BCR clones was observed in samples collected at 16 weeks after treatment (mean percentage FU2: 60%). Collectively, these results indicate that the postdepletion peripheral blood repertoire is mainly composed of highly mutated BCR clones. In addition, BCR clones with low mutation load in peripheral blood are markedly reduced 4 weeks after treatment, but it takes more time to see similar changes in synovial tissue.

### Rituximab treatment completely replaces the most dominant BCR clones in the repertoire

**Figure 3:**
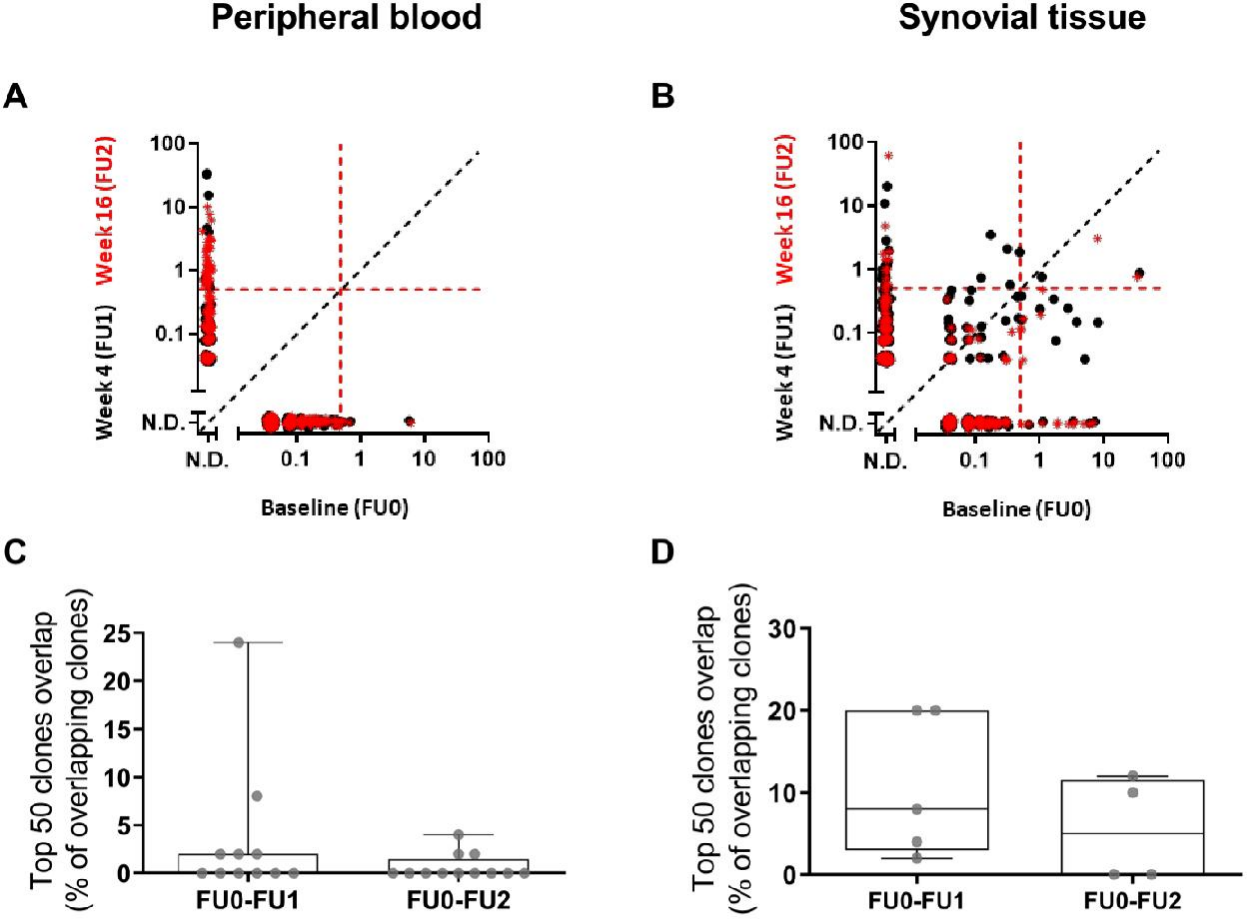
Clonal overlap in peripheral blood and synovial tissue BCR repertoire during treatment with rituximab. (A–B) Example of clonal overlap plots in the peripheral blood (A) and synovial tissue (B) BCR repertoire from one patient when comparing baseline with week 4 repertoire (black dots) or baseline with week 16 repertoire (red stars). Each symbol represents a unique BCR clone, and its frequency in the analysed repertoire is depicted on the x (baseline) and y (follow-up time point) axes as percentage of total reads. The red dotted lines indicate the 0.5% cut-off for dominant BCR clones, and the black dotted line indicates the plot diagonal. (C–D) Boxplot showing the clonal overlap within the top 50 clones of the pretreatment (FU0=baseline) and the post-treatment (FU1=week 4; FU2=week 16/24) BCR repertoires in peripheral blood (C) and synovial tissue (D). Boxplots show the median and 25th and 75th interquartile, error bars show the range, and single data points are depicted in grey. BCR, B cell receptor; N.D. not detected.

Compared with conventional cytometric techniques, BCR repertoire analysis offers the possibility to track individual BCR clones over time. Figure 3A shows an example of clonal overlap between the pretreatment BCR repertoire (FU0, x-axes) and the post-treatment BCR repertoires (FU1 and FU2, y-axes) of a rituximab-treated patient. The absence of shared BCR clones indicates that in this patient, all BCR clones present in peripheral blood prior to rituximab treatment cannot be retrieved in the post-treatment repertoires. To assess the qualitative changes in the BCR repertoire clonal composition induced by rituximab, we performed the clonal overlap analysis of the pretreatment and post-treatment BCR repertoires described above for all patients studied. The percentage of the overlapping top 50 clones (ie, how many identical BCR clones were shared within the 50 most dominant clones of the two repertoires in the comparison) was determined. Extremely low top 50 clonal overlap with the baseline repertoire was observed in peripheral blood at both time points after treatment (median (IQR): FU0–FU1: 0% (0%–2%); FU0-FU2: 0% (0%–1.5%)) (figure 3C), indicating that in nearly all patients the BCR clones present in peripheral blood before treatment could not be detected in the post-treatment repertoires. In contrast, a higher top 50 clonal overlap was observed when comparing synovial tissue obtained 4 weeks after treatment with baseline samples, with a subsequent strong decrease in top 50 clonal overlap 16 weeks after rituximab treatment (median (IQR): FU0–FU1: 8% (3%–20%); FU0–FU2: 5% (0%–11%)) (figure 3D). Together, these data show that rituximab treatment wipes out the dominant BCR clones from peripheral blood repertoire within the first 4 weeks of treatment and for up to 24 weeks after treatment. In synovial tissue the BCR repertoire was unaltered 4 weeks after treatment, but the clonal overlap was clearly decreased 16 weeks after treatment.

### Persistence of pretreatment dominant BCR clones in peripheral blood after 1 month of treatment associates with worse treatment response

**Figure 4:**
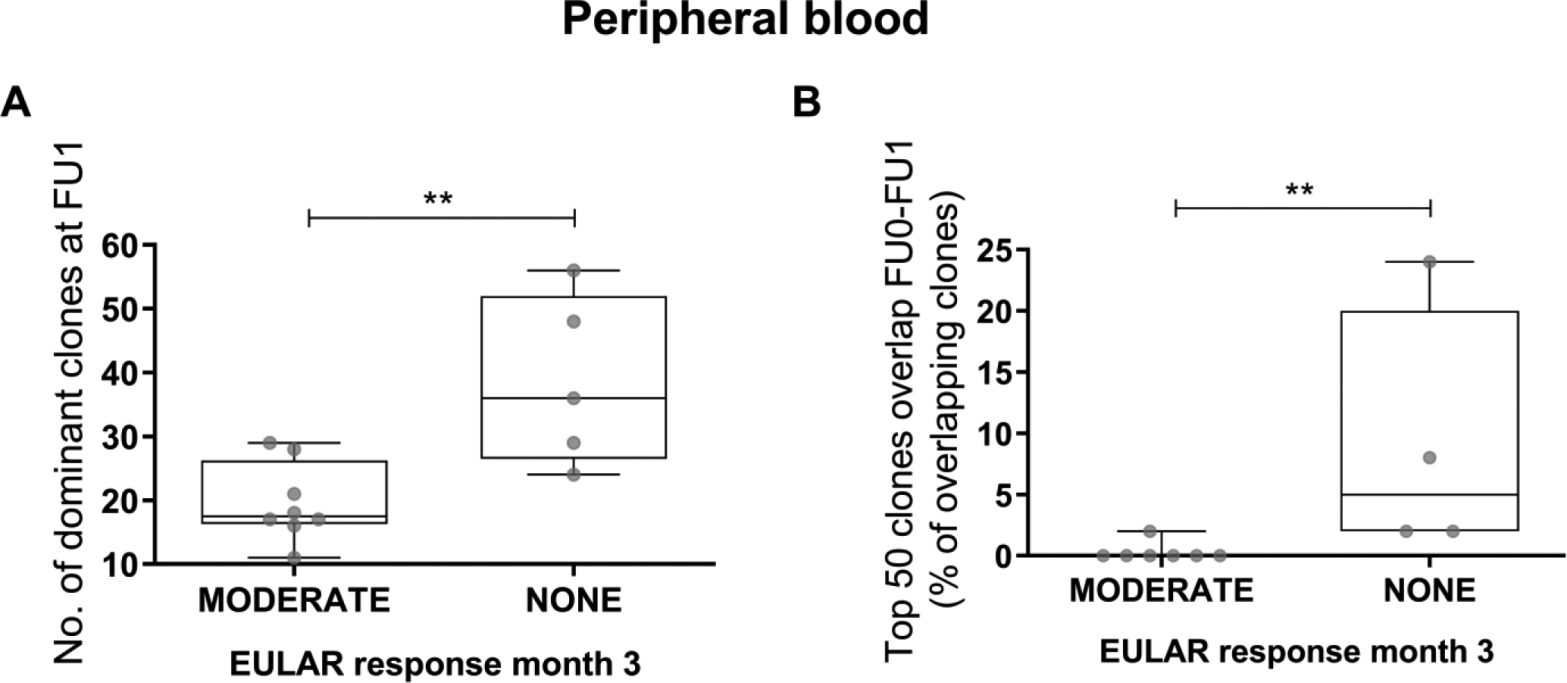
Correlation between peripheral blood BCR repertoire characteristics and clinical response at 3 months. (A) Boxplots showing the number of dominant BCR clones (clonal frequency >0.5%) in the peripheral blood repertoire at 4 weeks (FU1=week 4) after rituximab treatment in moderate and non-responder patients evaluated at 3 months. (B) Boxplots showing the clonal overlap within the top 50 clones of the pretreatment (FU0=baseline) and the first post-treatment (FU1=week 4) peripheral blood BCR repertoires in moderate and non-responder patients evaluated at 3 months. Boxplots show the median and 25th and 75th interquartile, error bars show the range, and single data points are depicted in grey (**p≤0.01 using Mann-Whitney test). BCR, B cell receptor; EULAR, European League Against Rheumatism.

Different biomarkers for response to rituximab have been proposed, but prediction of treatment response in the context of precision medicine remains a challenge.^7 12 19^ We therefore examined whether characteristics of the BCR repertoire before and after rituximab treatment could be predictive of treatment response. We analysed the differences between the baseline (FU0) and week 4 post-treatment (FU1) repertoire and correlated these to the patients’ clinical response evaluated 3 months after treatment using the European League Against Rheumatism (EULAR) response criteria.^20^ Paired baseline and week 4 peripheral blood samples were available for a total of 14 patients, of whom 5 were non-responders, 9 were moderate responders and none was a EULAR good responder at month 3.^12^ There were two predictors of non-response: first, non-responders at week 12 had an increased number of dominant BCR clones in the peripheral blood repertoire 4 weeks after treatment (FU1) compared with moderate responders (median (IQR): moderate responders: 18 (16–26); non-responders: 36 (27–52); p<0.01) (figure 4A). Second, non-responders showed a higher overlap of the top 50 clones between the week 4 and baseline repertoires compared with moderate responders (moderate responders: 0% (0%–0%); non-responders: 5% (2%–20%); p=0.0091) (figure 4B). Taken together, these findings indicate that persistence of the baseline repertoire after 4 weeks of rituximab treatment is associated with subsequent non-response.

## Discussion

In this experimental medicine study, we analysed the BCR repertoire to provide more insight into the effects of rituximab treatment on the peripheral blood and synovial tissue B cell compartments.

Our findings confirm and extend previous work showing that persistence of specific B cells is related to lack of clinical response to rituximab treatment in individual patients.^9^ Of interest, patients who do not achieve complete depletion of the BCR repertoire in peripheral blood within the first month of treatment do not achieve a clinical response after prolonged follow-up. These results indicate that early changes in peripheral blood could help to identify non-responders at an earlier time point, which would make it possible to switch patients who are unlikely to respond to rituximab treatment to other therapy.^19^ These observations are in line with previous work showing that residual memory B cells and therefore pathogenic immunological memory persists after rituximab treatment.^9 21^ This may help explain why a single course of rituximab, while clearly improving disease activity on the group level, does not cure RA.^3 22^

In addition, our findings raise the intriguing question as to which mechanisms are involved in the persistence and recurrence of specific BCR clones in individual patients. Future work addressing this question may pave the way for novel therapeutic strategies aimed at deep B cell intervention, for instance through innovative combination therapies.^23^ Rituximab-resistant BCR clones in peripheral blood are associated with treatment failure, as shown here, and memory B cells are the predominant repopulating fraction in patients with early relapse after B cell recovery.^8 24^ Why BCR clones may be resistant to rituximab treatment is not completely understood. Possible mechanisms include the presence of CD20-negative BCR clones (eg, plasma blasts), the presence of B cells that may have acquired prosurvival features or a relatively high influx of BCR clones into peripheral blood from the tissues where they are protected against cell death. The analysis of the BCR repertoire in synovial tissue based on the mutation load in the IGHV genes did indeed reveal the dominance of highly mutated BCR clones. This is in line with the notion that organised lymphoid aggregates, which contain mainly mature (and thus mutated) memory and plasma B cell subsets, are present in rheumatoid synovium.^25 26^ After rituximab treatment, a further increase in the highly mutated clonal fraction in the synovial tissue BCR repertoire is most likely a consequence of the reduction of BCR clones with low mutation load. This reduction could be attributed to a direct effect of rituximab on synovial tissue B cells, or could reflect reduced influx of immature B cells from the peripheral blood,^18 27–29^ a mechanism thought to be important in the perpetuation and in the onset of the clinical signs and symptoms of the disease.^29^

Our study has several weaknesses. First, the number of patients here analysed is low due to the requirement for repeated synovial biopsies. As a consequence, the observed correlation between early changes in peripheral BCR clonality and treatment outcome needs additional exploration to establish its test characteristics in larger cohorts with standard clinical practice. Furthermore, given the limited number of retrieved reads using the Roche platform, we focused on the dominant clones (>0.5% of the repertoire) since the sequence depth has only minor effects on their quantification (online supplementary figure S3). It should also be noted that our analysis is performed starting from RNA and should therefore be interpreted as a repertoire analysis of BCR clones, rather than an actual representation of the B cell repertoire on the DNA level. This is relevant when looking at the persisting, highly mutated clones on the RNA level that likely reflect (CD20-negative) plasma blasts and/or plasma cells. The presence of these cells is expected to increase the number of dominant clones, in line with our results. Although we did not detect B cells using conventional flow cytometry, we do not exclude the possibility that part of the BCR signatures does derive from B cells, which might be detectable by more sensitive flow cytometry techniques. Finally, our approach yields little information on the phenotype and specificity of the detected dominant BCR clones. Do these BCR clones encode rheumatoid factor and/or anticitrullinated protein specificity? In this context it would be of interest to investigate whether repertoire analysis also shows dominant BCR clones in seronegative patients with RA and whether the impact of rituximab on such dominant clones is identical.

In conclusion we have shown that in peripheral blood of RA patients, the postrituximab BCR repertoire is mainly composed of fewer but more dominant and more mutated BCR clones. In the synovial tissue, changes in BCR clonality are observed at later time points compared with peripheral blood, which could explain in part the relatively slow onset of clinical efficacy of rituximab treatment. Patients who do not respond to rituximab at 3 months show incomplete depletion of the baseline BCR clonal repertoire in peripheral blood within the first month of treatment, revealing a promising early predictive marker of clinical response.
