# Supplementary material for: Non-response to rituximab therapy in rheumatoid arthritis is associated with incomplete disruption of the B cell receptor repertoire
Source: Ann Rheum Dis. 2019 Jun 19;78(10):1339–45. doi: 10.1136/annrheumdis-2018-214898 (PMC6788876; doi:10.1136/annrheumdis-2018-214898)
Supplement: Supplementary data [file annrheumdis-2018-214898supp001.pdf]

Figure S1

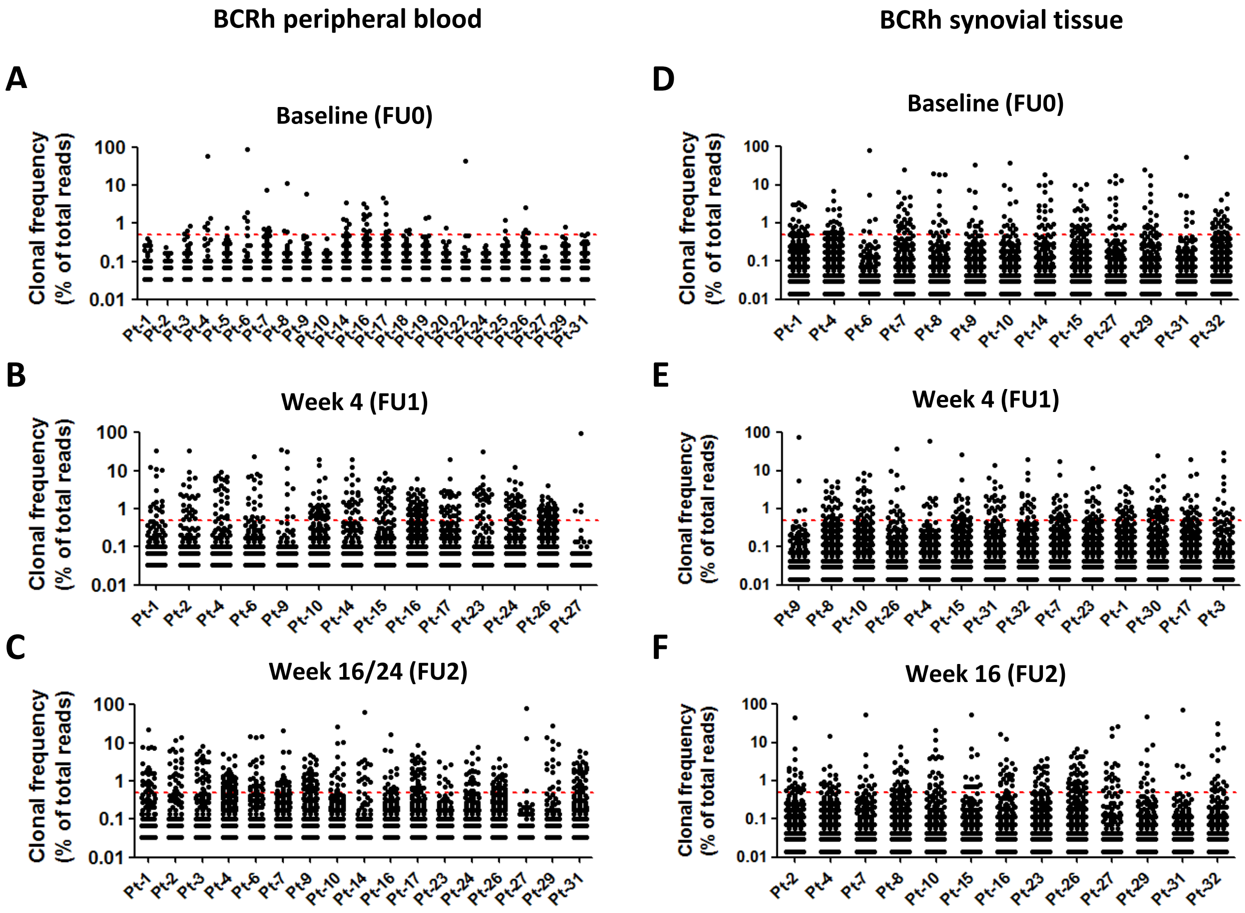

Figure S1: Scatter plots showing the clonal distribution in the peripheral blood BCR repertoire at baseline (A), at week 4 (B) and at week 16/24 (C) after rituximab treatment and in the synovial tissue repertoire at baseline (D), at week 4 (E) and at week 16/24 (F) after rituximab treatment. The red dotted line indicates the 0.5% cut-off for dominant BCR clones.

Figure S2

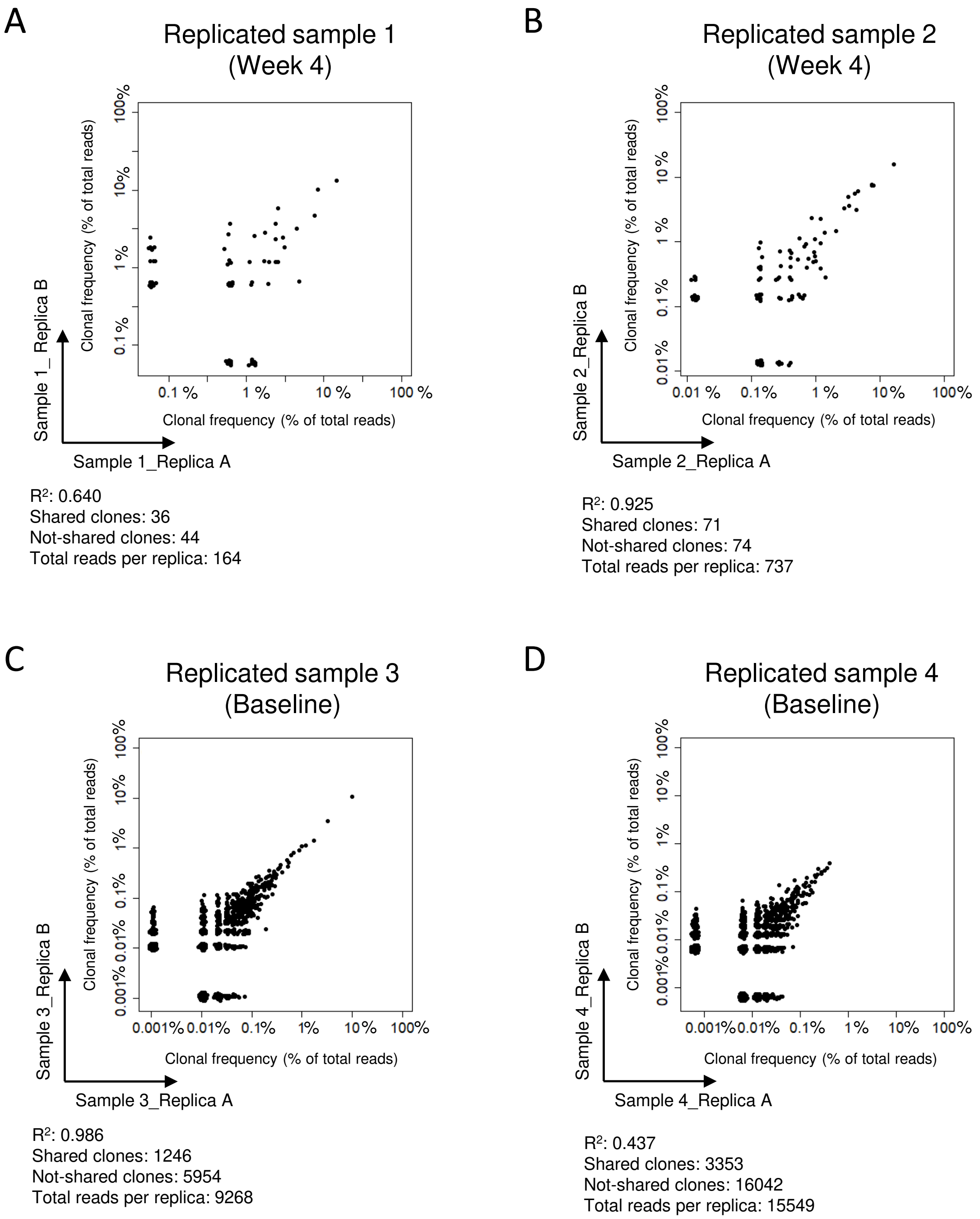

Figure S2: Scatter plots depicting the clonal overlap in two replicated 4 weeks post-rituximab treatment samples (A-B) and two replicated baseline samples (C-D). Each dot represents a single BCR clone and its frequency in each compared repertoire is depicted on the axes as percentage of total reads. For fair comparison, reads were randomly selected from the larger sample to attain equal total read numbers per replica (reported below each plot). The samples were amplified with the same primer set described in the manuscript but on a different sequencing platform (Illumina Miseq). Results of the evaluation of the clonal overlap are reported below the graphs as R^2 and number of shared and not-shared clones.

Figure S3

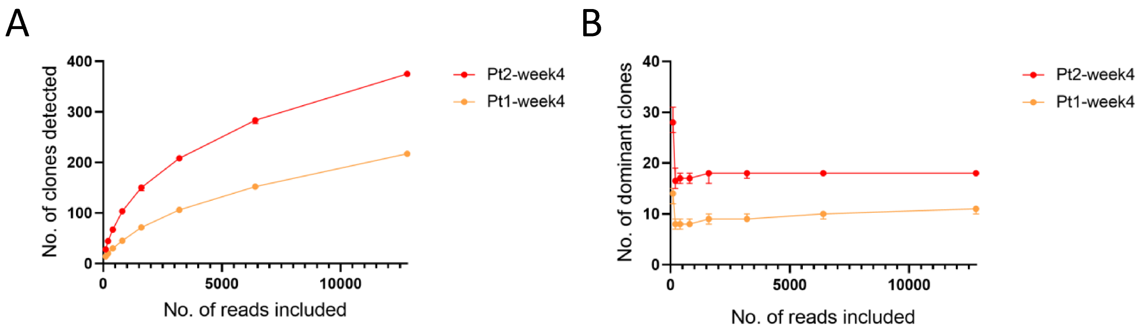

Figure S3. Line plot depicting the number of total clones (A) and the number of dominant clones (>0.5% of total reads) (B) in libraries of increasing size from two week 4 post-treatment samples from the manuscript cohort. While decreasing the read depth influences the number of total clones detected, the number of dominant clones remains stable above the 200 reads included in each sample.

Table S1

**DEMOGRAPHICS**	
Median age, years (range)	55 (22-75)
Female, no. (%)	18 (75%)
**DISEASE STATUS**	
Median disease duration, years (range)	12 (1-50)
IgM-RF positive, no. (%)	19 (79%)
Median IgM-RF, kU/L (IQR)	53 (23-113)
ACPA positive no. (%)	21 (86%)
Median ACPA, kU/L (IQR)	188 (61-1103)
Median DAS28 (IQR)	6 (5-7)
Median CRP, mg/L (IQR)	27 (11-72)
**MEDICATIONS**	
Median no. of previous DMARDs (IQR)	3 (2-5)
Median no. of previous BP (IQR)	2 (1-3)
Median methotrexate dosage, mg/week (IQR)	15 (10-25)
Median prednisone dosage, mg/day (IQR)	5 (0-10)

Table S1: Patients baseline characteristics. IgM-RF, IgM-rheumatoid factor; ACPA, anti-citrullinated protein antibodies; ESR, erythrocyte sedimentation rate; CRP, C-reactive protein; DMARDs, disease-modifying antirheumatic drugs; BP, biopharmaceutical.

Table S2

	CD19+ cells (10^9cell/L)	Phenotype (% of CD19+)			Library size (No. of reads)	Unique sequences
		IgD+ CD27-	IgD+ CD27+	IgD- CD27+		
**PERIPHERAL BLOOD**						
FU0_baseline (n=21)						
Pt-1	7	54.3	18.90	22.00	7477	5457
Pt-2	11	72.4	14.60	7.80	10308	8155
Pt-3	13	54.1	21.30	18.10	5295	4013
Pt-4	11	73.8	14.30	8.20	10601	2429
Pt-5	17	65.5	11.00	16.50	4079	3137
Pt-7	5	63.0	11.10	22.90	3878	1747
Pt-8	7	28.9	21.60	46.80	5335	3307
Pt-9	9	82.4	11.30	6.30	10845	7003
Pt-10	6	67.0	10.90	15.60	4748	3957
Pt-14	11	84.4	9.10	5.50	8953	3516
Pt-16	12	58.2	25.90	12.40	8261	3013
Pt-17	5	51.1	28.10	18.70	10445	3318
Pt-18	10	NA	NA	NA	9301	4270
Pt-19	7	72.6	12.90	11.50	9181	4745
Pt-20	14	74.5	24.80	0.50	9509	6319
Pt-24	14	71.4	10.80	13.80	10113	7319
Pt-25	14	70.9	16.00	11.50	7367	3878
Pt-26	9	44.1	10.00	36.00	3520	1972
Pt-27	20	78.5	7.80	10.30	10017	8350
Pt-29	5	NA	NA	NA	8896	4785
Pt-31	3	48.8	8.20	32.80	8020	4214

Table S2 (follow)

	CD19+ cells (10^9cell/L)	Library size (No. of reads)	Unique sequences
**PERIPHERAL BLOOD**			
FU1_week 4 (n=13)			
Pt-1	bdl	2462	81
Pt-2	bdl	4934	129
Pt-3	0.01	6023	216
Pt-6	Bdl	16545	419
Pt-9	bdl	19390	264
Pt-10	bdl	12632	353
Pt-14	bdl	4017	141
Pt-15	bdl	9661	328
Pt-16	bdl	11799	604
Pt-17	bdl	4870	407
Pt-23	0.01	5765	201
Pt-24	bdl	9623	493
Pt-26	bdl	11643	576
FU2_week 16 (n=13)			
Pt-1	bdl	12098	280
Pt-2	bdl	12509	274
Pt-3	bdl	4694	208
Pt-4	bdl	7297	436
Pt-6	bdl	5008	219
Pt-7	0.01	8226	554
Pt-9	bdl	5720	310
Pt-14	bdl	12416	189
Pt-17	bdl	8453	420
Pt-23	0.01	9178	2669
Pt-26	bdl	7284	642
Pt-29	bdl	8320	242
Pt-31	0.01	6872	315
FU2_week 24 (n=3)			
Pt-10	bdl	10587	389
Pt-16	0.02	13454	2135
Pt-24	0.04	10312	1500

Table S2 (follow)

	CD19+ cells (cells/mm³)	Library size (No. of reads)	Unique sequences
**SYNOVIAL TISSUE**			
FU0_baseline (n=9)			
Pt-1	136	6342	1563
Pt-4	0	3192	540
Pt-7	197	7274	647
Pt-8	229	4762	432
Pt-9	135	5802	845
Pt-10	0	5046	445
Pt-14	0	2558	173
Pt-15	0	5275	633
Pt-16	53	3362	368
FU1_week 4 (n=9)			
Pt-1	53	9868	1537
Pt-3	101	5467	436
Pt-4	55	6911	517
Pt-7	80	5629	938
Pt-10	115	3310	384
Pt-15	19	6825	771
Pt-17	0	4573	536
Pt-23	777	4078	934
Pt-26	0	15737	163
FU2_week 16 (n=6)			
Pt-7	763	5427	550
Pt-8	0	3338	625
Pt-10	67	5260	408
Pt-15	0	3647	235
Pt-23	87	4906	1068
Pt-26	0	3182	265

Table S2: List of patients' samples sequenced for this study divided by timepoint and tissue origin including initial amount of CD19+ B cells (bdl = below detection limit) [11, 13] and number of total and unique reads retrieved after sequencing. For baseline peripheral blood samples, percentage of naïve (IgD+CD27+), transitional (IgD+CD27+) and memory (IgD-CD27+) B cells are reported [11].
